# Sleep monitoring in mental health goes digital

**DOI:** 10.1038/s43856-021-00050-y

**Published:** 2021-11-19

**Authors:** Katharine Barnes

**Affiliations:** Communications Medicine, https://www.nature.com/commsmed

## Abstract

People with psychiatric disorders often experience sleep problems. A recent post hoc cross-sectional study in *PLoS Medicine* used movement readings from participants’ wrists to assess their sleep and then investigated associations with several psychiatric disorders.

Polysomnography, which records brain waves, blood oxygen, heart rate, breathing and movement, has traditionally been used to monitor sleep. However, this requires specialist equipment and is therefore not amenable to simultaneously monitoring the sleep of large numbers of people at home. Self-reporting of the quality and duration of sleep is often used instead, but is known to be less accurate. Recently wrist-worn devices have enabled movement patterns to be quantified whilst normal activities are being undertaken, and such accelerometer readings can be used to monitor sleep.

Whilst it has been shown that poor sleep is both a causal risk factor and common in people with many psychiatric disorders, most studies have focused on people experiencing particular disorders. Wainberg et al.^1^ investigated associations between sleep and diagnoses of schizophrenia spectrum disorders, bipolar disorder/mania, major depressive disorder and anxiety disorders. In contrast to many prior studies, participants were not selected for study based on known inpatient psychiatric diagnoses but were representative of the wider population. Over 80,000 participants from the UK Biobank were provided with an accelerometer and asked to wear it on their dominant wrist for 7 days. Ten sleep measures were assessed, including bedtime, wake-up time, sleep duration, sleep efficiency, number of sleep periods, naps and variability in sleep patterns.

Similar sleep pattern differences were present across all the disorders studied, generally with the disorders associated with reduced sleep quality rather than changes in sleep duration. Reduced sleep quality was also associated with polygenic risk scores derived from genome-wide association studies for major depression, bipolar disorder and schizophrenia. Whilst the effects tended to be small, participants were not necessarily experiencing symptoms of the disorder when their sleep was assessed, since these were lifetime diagnoses.

**Figure:**
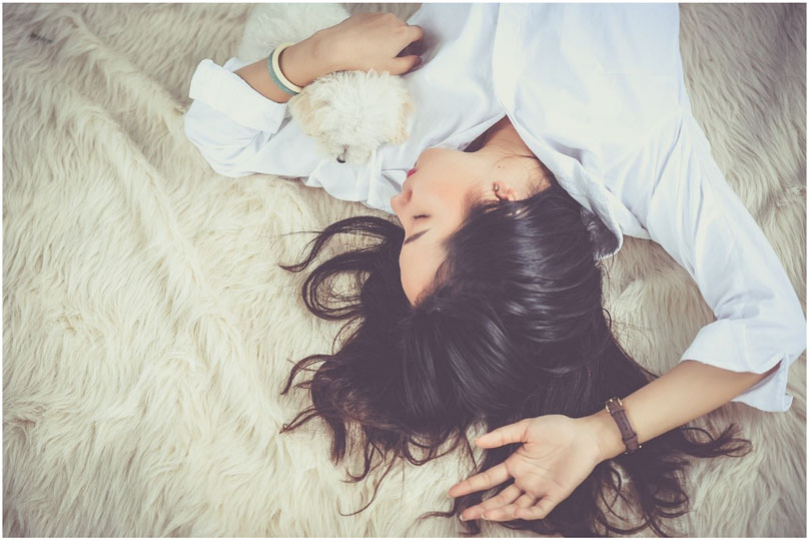
Image by Jess Foami from Pixabay.

A secondary analysis considered 6 sleep properties self-reported at baseline assessment. Some associations were seen between these sleep properties and the inpatient psychiatric diagnoses and psychiatric polygenic risk scores, despite the different assessment methods and the baseline assessment having occurred 5 years earlier. Comparisons were also made between self-reported and accelerometry-derived sleep measurements, with associations seen between sleep duration, naps and wake up times. Self-reported chronotype was also associated both with accelerometry-derived bedtime and wake-up times.

The initial analysis was undertaken on data from self-reported white participants, who are the majority of the participants in the UK biobank. However, a replication analysis of a cohort of 2692 self-reported non-white participants found similar associations. Males and females were also confirmed to have similar sleep alterations across lifetime psychopathologies.

Whilst relatively high median sleep efficiency, low wake time after sleep onset, and long sleep bout durations were obtained in this study compared to prior polysomnography-based studies, it was nevertheless able to assess the normal sleep patterns of vast numbers of participants, using a potentially less disruptive measure. Such wearable devices could provide a scalable way to objectively measure sleep across many thousands of individuals undertaking their normal behaviours, facilitating studies investigating the impact of sleep on development of other disorders.
